# Valid Evidence for Diagnosis and Treatment of Infections in the Intensive Care Unit: Beyond Randomized Control Trial Study Design (Trial Emulation and Machine Learning)

**DOI:** 10.3390/jcm11133600

**Published:** 2022-06-22

**Authors:** Erlangga Yusuf, Frits R. Rosendaal

**Affiliations:** 1 Department of Medical Microbiology and Infectious Diseases, Erasmus University Medical Center, 3015 GD Rotterdam, The Netherlands; 2 Department of Clinical Epidemiology, Leiden University Medical Center, 2300 RC Leiden, The Netherlands; f.r.rosendaal@lumc.nl

## 1. Infection in Critically Ill Patients and Randomized Control Trial

Infection in critically ill patients is an important problem. Half of patients admitted to the intensive care unit (ICU) have suspected or proven infection, and 30% of them die [1]. Not surprisingly, the use of antibiotics in the ICU is high. About 70% of ICU patients receive at least one antibiotic, either for prophylactic or therapeutic purposes. The frequency of infectious disease in the ICU has not changed in the last three decades, as shown in consecutive (European and Extended) Prevalence of Infection in Intensive Care (EPIC) studies in 1995, 2007 and 2017 [1,2,3]. These data suggest that the diagnostics and treatment of critically ill patients still need to be improved.

To achieve this, studies with rigorous designs that ask the right questions and are connected to real practice, collect the correct data in representative populations and perform appropriate analyses are needed. Especially due to its ability to minimize confounding factors, the randomized control trial (RCT) is considered the most reliable study design for generating clinical evidence. However, this design also suffers from drawbacks, of which the most important ones are lack of generalizability and a time-consuming process, which make it less than optimal for accelerating the discovery of evidence regarding the best therapeutic option to diagnose and treat critically ill patients. 

RCT is expensive to perform. A systematic review has showed that the median cost per recruited patient in an RCT was USD 409 and that the cost to run an RCT may be as high as USD 612 million [4]. The inclusion of patients often takes a long time, and often, the target sample size is not reached. Furthermore, patients included in an RCT are often different from patients in routine practice due to an abundance of exclusion criteria, which leads to the inclusion of mainly patients with few co-morbidities and good adherence to therapy. For example, a study showed that a significant proportion of patients (up to 40%) who received a vitamin K antagonist and were admitted to hospitals due to bleeding would have been excluded in the clinical trials on which the indication for the vitamin K antagonist is based. They would have been excluded mostly because of comorbidities. However, in routine practice, these multimorbid patients are exactly those who are at highest risk of complications of using a vitamin K antagonist (i.e., bleeding) [5]. In the ICU setting, a similar example is that patients with intraabdominal infections who are intubated often also have pneumonia but are excluded from trials on intraabdominal infection. This may result in a new antibiotic appearing effective in an RCT, whereas in real practice, the effect is diluted since the patients are different. Moreover, due to its design, an RCT can mostly only answer one or two clinical questions, while new treatments may emerge rapidly. The most common RCT question in the ICU is whether a new antibiotic is effective. To answer this question, a new antibiotic is compared with another well-established antibiotic (since comparisons with placebos would be unethical). In the case of an RCT on new antibiotics, the objective usually is to show non-inferiority, i.e., to show that the new antibiotic is ‘not unacceptably worse’ than the current standard therapy (as reviewed in this Special Issue regarding new antibiotics against Gram-negative bacteria [6]). This approach is logical, but it has some drawbacks. As the term suggests, it uses a ‘non-inferiority’ limit and this limit is often arbitrary. Furthermore, even when the new antibiotic is not inferior to the well-established antibiotic, other aspects such as cost and adverse events should also be demonstrated. It is certainly not anyone’s intention to use new antibiotics that are not worse than the standard therapy but at the same time more expensive.

### Beyond Randomized Control Trials

Alternative study designs may provide valid evidence for clinical questions in ICU patients such as observational studies and pragmatic clinical trials. These studies are often categorized as real world data [7]. An observational study uses data that have been collected in routine clinical practice, i.e., real life. Its population is not selected and is heterogeneous. Critically ill patients with intraabdominal infections and comorbidities may be included, and they may differ in APACHE II score, mental status, renal clearance and adherence to treatment protocol. These are the patients who will potentially receive the treatment in actual practice. Moreover, since the data are already collected for clinical purposes, this type of study is low-cost to perform compared with a RCT and has fewer ethical constraints. Due to its real-life characteristics, this type of study is useful for assessing complex therapies, for example, in the ICU setting. In contrast to an RCT, which only compares a treatment with another, real-life studies can integrate antibiotics, the use of mechanical ventilation and inotropic use. However, as all advantages have their disadvantages, these real-life study features may also be considered weaknesses. Due to the heterogeneous patient population, a study may fail to detect an overall difference in treatment effects and since data are not collected comprehensively, missing data may be a problem, not to mention that doctors may choose certain treatments based on their experience and that severely ill ICU patients may be monitored more closely and may receive other therapeutic regimes (e.g., antibiotic combinations or the addition of antifungal drugs) than less ill patients. These features will introduce ‘confound by indication’.

Surely these issues matter, but they can be overcome and improved. When more studies are published, systematic reviews and meta-analyses can be conducted to increase the power to detect differences in treatment effects. Possible confounders may be statistically adjusted (this statistical adjustment cannot remove bias and cannot remove confounding variables that are not measured) [8]. To improve the quality of observational studies, (R)CT principles can be applied for observational study design in the so-called target trial emulation [9,10]. Specifying the target trial’s eligibility criteria, treatment strategies, assignment procedures, follow-up period, outcomes, causal contrast of interest (i.e., intention-to-treat or per-protocol) and analysis plans can be improved [9]. This approach reduces bias and improves the precision of estimates in contrast to the straightforward use and analysis of observational data, but it is surely more complex.

To make further use of big observational data, applying machine learning (i.e., systems that are able to automatically learn and improve from experience without being explicitly programmed [11]) has great potential. Algorithms can be developed to automatically extract rules from observational data, for example, on which antibiotic and which other support measures are needed to identify patients with good clinical outcomes. Every time that new data are collected, the algorithm will be improved.

A comment should also be made on ‘pragmatic’ RCTs. This type of trial is conducted in a manner resembling situations in clinical practice to compare the effectiveness of interventions in a way that is applicable to decision makers [12]. Adding ‘pragmatic’ to the term RCT is increasingly used in medical publications, despite many of those studies often not having any ‘pragmatic’ characteristics. A pragmatic RCT is aimed at identifying which available intervention is better, with a primary endpoint that is patient-centered. It should mimic the real world (i.e., the number of tests, procedures and visits) and should be run in several sites (to ensure heterogeneous sample of subjects), and its analysis should focus on the ‘intention to treat’.

## 2. Conclusions

It is hard to deny the contribution of RCTs in adding clinical evidence. However, due to its shortcomings and to make use of the improvements from the 21st century, other study designs may offer accelerations in the discovery of valid evidence for answering complex clinical questions in complex patients such as those who are critically ill. Those study designs include observational studies or trial emulations. Applying machine learning in observational data may be the next level to observational studies.
